# Glucose Starvation Boosts *Entamoeba histolytica* Virulence

**DOI:** 10.1371/journal.pntd.0001247

**Published:** 2011-08-02

**Authors:** Ayala Tovy, Rivka Hertz, Rama Siman-Tov, Sylvie Syan, Daniela Faust, Nancy Guillen, Serge Ankri

**Affiliations:** 1 Department of Molecular Microbiology, The Bruce Rappaport Faculty of Medicine, Technion, Israel; 2 Institut Pasteur, Unité Biologie Cellulaire du Parasitisme, Paris, France; 3 Inserm, U786, Paris, France; Jawaharlal Nehru University, India

## Abstract

The unicellular parasite, *Entamoeba histolytica*, is exposed to numerous adverse conditions, such as nutrient deprivation, during its life cycle stages in the human host. In the present study, we examined whether the parasite virulence could be influenced by glucose starvation (GS). The migratory behaviour of the parasite and its capability to kill mammalian cells and to lyse erythrocytes is strongly enhanced following GS. In order to gain insights into the mechanism underlying the GS boosting effects on virulence, we analyzed differences in protein expression levels in control and glucose-starved trophozoites, by quantitative proteomic analysis. We observed that upstream regulatory element 3-binding protein (URE3-BP), a transcription factor that modulates *E.histolytica* virulence, and the lysine-rich protein 1 (KRiP1) which is induced during liver abscess development, are upregulated by GS. We also analyzed *E. histolytica* membrane fractions and noticed that the Gal/GalNAc lectin light subunit LgL1 is up-regulated by GS. Surprisingly, amoebapore A (Ap-A) and cysteine proteinase A5 (CP-A5), two important *E. histolytica* virulence factors, were strongly down-regulated by GS. While the boosting effect of GS on *E. histolytica* virulence was conserved in strains silenced for Ap-A and CP-A5, it was lost in LgL1 and in KRiP1 down-regulated strains. These data emphasize the unexpected role of GS in the modulation of *E.histolytica* virulence and the involvement of KRiP1 and Lgl1 in this phenomenon.

## Author Summary

During infection, pathogens are exposed to different environmental stresses that are mostly the consequence of the host immune defense. The most studied of these environmental stresses are the response of pathogens to nitric oxide and to hydrogen peroxide, both produced by phagocytes. In contrast, the overall knowledge about the response of pathogens to metabolic stresses is scanty. Amebiasis is caused by the unicellular protozoan parasite *Entamoeba histolytica,* and has a worldwide distribution with substantial morbidity and mortality. During its journey in the host, the parasite is exposed to the host immune system and to variations in nutrient availability due to the host nutrition status and the competition with the bacterial flora of the large intestine. How *E. histolytica* responds to glucose starvation (GS) has never been investigated. Here, the authors report that the parasite virulence is boosted by GS. Paradoxically, two well accepted virulence factors, the amoebapore A and the cysteine protease A5 are less abundant in the glucose-starved parasites. This Accordingly, these proteins are not required for the boosting of the *E. histolytica* virulence, in contrast to KRiP1 and LgL1 that seem to be involved in this phenomenon.

## Introduction

Amebiasis is a parasitic infection that is caused by the unicellular protozoa, *Entamoeba histolytica*. The disease has a worldwide distribution with substantial morbidity and mortality, and is one of the three most common causes of death from parasitic disease [1]. The main clinical manifestations of amebiasis are colitis and liver abscesses. Pathogenesis of *E. histolytica* involves adherence, penetration into host tissues, and destruction of host cells. These processes are mediated by the ameba key virulence factors galactose/N-acetylgalactosamine (Gal/GalNAc) lectin, amoebapore (AP) and cysteine proteinases (CP). Host cell destruction is initiated upon trophozoites binding of the target cells. An important molecule involved in this process is the galactose/N-acetylgalactosamine-inhibitable lectin [2]. This molecule is composed of two subunits; the 170 kDa heavy chain, which is responsible for the cell and carbohydrate binding activity of the lectin complex, and the 31–35 kDa light chain that has a structural role and participates in membrane anchoring of the complex [3]. It is believed that adherence of the parasite to the host gut cells is followed by the release of APs, which are a family of at least three small peptides capable of forming pores in lipid bilayers [4]. Other factors that play an important role in the *Entamoeba* pathogenesis are the CPs. These enzymes are released by the parasite, to disrupt the intestinal mucus and the epithelial barrier and to facilitate the tissue penetration by the trophozoites [5], [6]. The life cycle of the parasite consists of two stages: the infective cyst and the invasive trophozoite. During its progression through its life cycle in the host, the parasite is exposed to different environmental stresses which are the direct consequence of the host immune defence, or metabolic modifications and changes in the bacterial intestinal flora [7]. Whereas the physiological and molecular changes in *E. histolytica* following their exposure to oxidative and nitrosative stress(es) [8], [9], [10], [11], heat shock [12], [13], and bacterial flora [14], [15], [16] have been well investigated over the past ten years, information about the effects of metabolic stress in this parasite is lacking. *E. histolytica* relies solely on glycolysis and fermentation and lacks the tricarboxylic acid cycle and the mitochondrial electron chain reactions. Energy is mainly obtained from glucose fermentation, producing carbon dioxide, acetate and ethanol. Glucose starvation (GS) is a widely studied metabolic stresses in pathogens. It has been investigated in the malaria parasite *Plasmodium falciparum*. Interestingly, the PfEMP (*var*) genes, key components in malaria pathogenesis, account among the genes up-regulated by GS [17]. Recently, we found that in *E. histolytica,* GS leads to the accumulation of the glycolytic enzyme enolase in the nucleus and to the inhibition of the DNA and tRNA methyltransferase 2 (Dnmt2) nuclear activity [18]. In addition, GS triggers the *in vitro* differentiation of *Entamoeba invadens* trophozoites into cysts [19], a finding potentially relevant for *E. histolytica*. Here, we describe data on the effects of GS on the physiology of the parasite. We report that GS is a positive regulator of *E. histolytica* virulence. To the best of our knowledge, this is the first evidence that supports a role of a metabolic stressor in the modulation of *E.histolytica* virulence.

## Methods

### Growth of *Entamoeba histolytica*

Trophozoites of the *E. histolytica* strain HM1:IMSS were grown under axenic conditions in Diamond's TYI-S-33 medium at 37°C. Trophozoites in the exponential phase of growth were used in all experiments. For the GS assays, trophozoites were washed three times with PBS, and then incubated for 12 hours in glucose-free Diamond's TYI-S-33 medium. Recovery was done by adding 1% glucose to the GS parasites for an additional 12 hours in the same medium.

### Construction of sense and antisense KRiP1

For the construction of the antisense plasmid, KRiP1 was amplified by PCR from genomic DNA using the primers KRIP S and KRIP AS (Table1). The resulting 1500 bp PCR product was cloned into the pGEM-T Easy vector (Promega) to give pGEM-KRiP. For the construction of the control sense KRiP1 plasmid, KRiP was amplified from pGEM-KRiP with the primers KRiP Bgll II and KRIP AS and subcloned into the pGEM-T Easy vector (Promega) to give pGEM-sense KRiP. Next, BglII/NotI-digested pGEM-KRiP and pGEM-sense KRiP were subcloned into BglII/NotI-digested pSA8 plasmid [20]. The resulting plasmids contain the complete coding region of KRiP1 in the antisense or in the sense orientation, respectively, between 5′ and 3′ untranslated regions of the *E.histolytica* gene coding for ribosomal protein RP-L21 [20]–[21].

**Table 1:** Oligonucleotides used in this study.

Accession number	Primer name	Sequence
X70851	AP-A 5′	AAACATATCTTACAAACAATC
X70851	AP-A 3′	GAGTTTATCAATTCCAAAAT
EHI_130700	Enolase 922 5′	AATAAATTCACTGTTGAACATGGTAAT
EHI_130700	Enolase 3′	TTAAGCAGTTGAATTTCTCCAGTT
X91644.2	CP-A5 sense	CTGAATATTGGATTGTTAGAAATTCAT
X91644.2	CP-A5 anti sense	AAGCATCAGCAACCCCAACTGGA
M96024	LgL1 sense	CTCTAGAGACATCTAAAATATTGTC
M96024	LgL1 anti sense	GGAGATCTGAATTGCATAACTTGTGTACAT
AF291721	URE3-BP sense	ACTTCTCTCTGTTATTACACAGACAGACTTT
AF291721	URE3-BP anti sense	ACCAAAAATGAAATTTTTATTTATTC
EHI_091570	HSP bis70 5′	TAAGAAGAGTGGCAAAAAGAGTATTG
EHI_091570	HSP 70 3′	TGTATTCTACTTCAATTAATGGTTTAT
EHI_096350	KRiP S	ATGGTACCATGTTTATCTTTTATTA
EHI_096350	KRiP AS	ATAGATCTTTAAATCTTAACTTGATT

### Measurement of cytopathic activity

The rate of cultured HeLa cell monolayers destruction by trophozoites that were grown in either control or glucose-free medium was determined using a previously described protocol [22]. Briefly, *E. histolytica* trophozoites (2.5×10^5^ or 10^5^ per well) were incubated with HeLa cell monolayers in 24-well tissue culture plates at 37°C for 60 minutes. The incubation was stopped by placing the plates on ice and unattached trophozoites were removed by washing the plates with cold Phosphate Buffer Saline (PBS). HeLa cells remaining attached to the plates were stained with methylene blue (0.1% in 0.1 M borate buffer, pH 8.7). The dye was extracted from the stained cells by 0.1 M HCl, and its color intensity was measured spectrophotometrically at OD_660_.

### Hemolytic activity

The hemolytic activity of trophozoites that were grown in either control or glucose-free medium was determined using a previously described protocol [22]. Briefly, Human red blood cells were collected in heparin and then washed three times in PBS. The assay was performed by mixing 2.5×10^5^ trophozoites that had been grown in either control or glucose-free medium with 5×10^8^ red blood cells in 1 ml PBS at 37°C for 60 min. Samples of these mixtures were taken at 30 and 60 minutes. The cell suspension was rapidly sedimented (6000 rpm for 5 seconds), and the amount of hemoglobin in the supernatant was determined spectrophotemetrically at OD_570_.

### Adhesion assay

The adhesion of trophozoites that were grown in either control or glucose-free medium to an HeLa cell monolayers was measured using a previously described protocol [23]. Briefly, trophozoites (2×10^5^) were added to wells that contained fixed HeLa monolayers in 1 ml of Dulbecco's modified Eagle's medium (DMEM) without serum, and incubated at 37°C for 30 minutes. The number of adherent trophozoites was determined by counting under a light microscope the trophozoites that remained attached to the HeLa cells after gentle decanting (twice) of the non-adherent trophozoites with warmed (37°C) DMEM.

### Transwell migration assays

Transwell migration assays were performed in 5 mm transwell inserts (8 µm pore size Costar) suspended by the outer rim within individual wells of 24-well plates using a previously described protocol [24]. Briefly, *E. histolytica* trophozoites that were grown in either control or glucose-free medium, were first washed three times in Diamond's TYI-S-33 medium without serum, and then suspended at a concentration of 2×10^5^ trophozoites/ml in serum-free medium. A 500-µl aliquot of the suspension was then loaded into the upper chamber of the transwell inserts, which were then placed in anaerobic bags (Mitsubishi Gas Chemical Company, Inc., Tokyo, Japan), and incubated at 37°C for three hours. At the end of the incubation, the inserts and media were removed, and trophozoite migration was determined by counting the number of trophozoites that were attached to the bottom of the well.

### Viability assay under heat shock and oxidative stress

Trophozoites (1×10^6^) were exposed to a temperature of 42°C or to 2.5 mM in hydrogen peroxide (H_2_O_2_). After 1, 1.5, 2 and 2.5 hours of incubation, an aliquot of the culture was stained with eosin (0.1% final concentration), and the number of living trophozoites were counted in a counting chamber under a light microscope (Bausch and Lomb).

### Stable isotope labeling by peptide dimethylation for quantitative proteomic analysis

*Entamoeba histolytica* trophozoites (10^7^) that were grown in either control or glucose-free medium for 12 hours were harvested and lysed in 300 µL 1% octyl β-D-glucopyranoside (Sigma-Aldrich) on ice for 20 minutes with regular shaking. Crude extracts were centrifuged at 13000 rpm at 4°C for 10 minutes, supernatants were removed, and the protein amounts of in the lysate were quantified using the Bradford method [25]. Usually 4 mg of protein were recovered and 100 µg of total cell lysate was used for the labeling in two independent experiments. Urea (8M) was then added to the proteins followed by their reduction with 3 mM DTT at 60°C for 30 minutes. Trypsinization of proteins was carried out overnight at 37°C in 10 mM ammonium bicarbonate containing modified trypsin (Promega) at a 1∶50 enzyme-to-substrate ratio, overnight at 37°C. A second step of trypsinization was done by adding another portion of trypsin and incubating at 37°C for 4 hr. The tryptic peptides were desalted using C18 tips (Harvard Inc.), dried and resuspended in 50 mM Hepes (pH 6.4). Labeling by dimethylation was done in the presence of 100 mM NaCBH_3_ (Sterogene). Peptides from the control samples were labeled with light formaldehyde (35% Frutarom) and peptides from glucose starvation samples were labeled with heavy formaldehyde at a final concentration of 200 mM (20% w/w, Cambridge Isotope laboratories). After 1 hour incubation at room temperature, the pH was raised to 8 and the reactions were further incubated for 1 hour. Neutralization was done with 25 mM ammonium bicarbonate for 30 minutes and equal amounts of the “light” and “heavy” peptides were mixed, cleaned on C18 tips and re-suspended in 0.1% formic acid. The combined labeled peptides were separated in an on-line two-dimensional chromatography (MuDPiT). First the peptides were loaded on a 15 mm of BioX-SCX column (LC Packing) and eluted with 10 salt steps of 0, 30, 60, 80,100, 120, 160, 200, 300, 500 mM ammonium formate in 5% ACN and 0.1% formic acid, pH 3. The eluted peptides were further resolved by capillary reverse-phase chromatography (20 cm fused silica capillaries, J&W self-packed). The peptides were eluted using a 125 min gradient (5% to 40% acetonitrile containing 0.1% formic acid) followed by a wash step of 95% acetonitrile for 15 min. The flow rate was about 0.25 µl/min and the peptides were analysed using an Orbitrap mass spectrometer (Thermo-Fischer). Protein identification was performed at the Smoler Proteomics Center, Technion (http://biology.technion.ac.il/proteomics/index.htm). Mass spectrometry (MS) was done in a positive mode using repetitively full MS scan followed by collision induced dissociation (CID) of the 7 most dominant ions selected from the first MS scan. The MS data were analyzed and compared using the Sequest 3.31 (Thermo). Identification of proteins with significant changes in their abundance level was based on searching with the ProtScore program, with a cut-off at 2.0 fold, searching in the *E. histolytica* genome NZ_AAFB00000000 part of the NR-NCBI data base.

### Fractionation of trophozoites

Nuclear and cytoplasmic fractions of *E.histolytica* trophozoites were prepared using a previously described protocol [21]. Proteins were separated on 12% polyacrylamide SDS-PAGE gels, and then transferred to a nitrocellulose membrane. Membranes were stained with Ponceau S (Sigma) to verify the efficiency of the transfer. The blots were then blocked with 3% skim milk in PBS, and then incubated with a monoclonal antibody against actin (1∶1000, Santa-Cruz), a polyclonal antibody against *E. histolytica* amoebapore A (Ap-A) (1∶500) (kindly provided by Prof. Mirelman, Weizmann Institute, Israel), and a polyclonal antibody against *E. histolytica* KRiP1 (1∶1000) raised in this work by three immunizations of two rabbits with a unique synthetized peptide (amino acids 82 to 108, EQSTKAPAGDKVINLD). After incubation with the first antibody, the blots were then incubated with a secondary antibody (1∶10000) (horseradish peroxide-conjugated goat anti-rabbit) antibody, Jackson ImmunoResearch), and immune complexes were detected by enhanced chemiluminescence.

### Preparation of membrane fraction

Trophozoites were harvested by centrifugation, washed 3 times in PBS, resuspended in 300 µl 100 mM NaCl and subjected to four cycles of freezing at -70°C and thawing. The samples were centrifuged at 800 g for 15 minutes and supernatants were collected and centrifuged at 10000 g for 1 hour. The Pellets were resuspended in 15 µl PBS.

### RT-PCR analysis

Total RNA was extracted from trophozoites using a TRI-reagent solution (Sigma). Reverse transcription was performed with the EZ-First Strand cDNA Synthesis Kit for RT-PCR (Biological Industries, Beit Haemek, Israel), according to the manufacturer's instructions. The primers that were used to amplify Ap-A, LgL1, CP-A5, URE3-BP, enolase encoding genes and rDNA are listed in Table 1. The PCR products were resolved on 1% agarose gels, and then stained with 0.5 µg/ml ethidium bromide (aMReSCO). Densitometry analysis of the stained PCR products was done using the TINA software (http://www.tina-vision.net).

### Northern blot analysis

Total RNA was prepared using the RNA isolation kit TRI Reagent (Sigma). RNA (10 µg) was size-fractionated on a 4% polyacrylamide denaturing gels containing 8 M urea and subsequently blotted to a GeneScreen membranes (NEN Bioproducts, Boston, MA). Hybridizations under stringent conditions were carried out overnight at 65°C in hybridization buffer (0.5 M Na-phosphate (pH 7.2), 7% SDS, 1 mM EDTA and 50 µg per ml hybridization buffer of salmon sperm DNA with the following DNA probes (0.004 µg/ml): Ap-A, CP-A5, URE3-BP and HSP 70. The membrane was then washed at 65°C for 20 min with washing buffer 1 (5% SDS, 40 mM Na-phosphate (pH 7.2), and 1 mM EDTA), followed by three washes of 30 min each wash at 65°C with washing buffer 2 (1% SDS, 40 mM Na-phosphate (pH 7.2), and 1 mM EDTA). Probes were randomly labeled with P^32^ dCTP (Amersham) using a DNA labeling kit (Biological Industries).

### Hepatic abscess formation

Animal handling and experimentation were conducted according to the European Union and the Pasteur Institute approved protocols. Four-week-old male Syrian golden hamsters (*Mesocricetus auratus*), with a weight ranging from 90 to 100 g, were inoculated by the intraportal route [26], [27] with 10^6^ sense or anti-sense KRiP1 trophozoites. Two independent experiments were performed, with 3 animals for each amoeba type per experiment; thus, a total of 12 hamsters were used for this evaluation. (Six animals by experiment and conditions were used; in total, 12 hamsters were included in this evaluation. Treatment of animals and surgical procedures were done according to published methods. From 7 days post-infection, hamsters were sacrificed, livers removed). Livers were isolated 7 days after infection and treated for histological analysis. Serial (five-micron) lobe sections were stained with Harris' haematoxylin, periodic acid-Schiff reagent. Immunohistochemistry was performed on sections from different livers using an anti-Gal/GalNAc lectin monoclonal antibody CD6 (a total of 31 tissue sections were analysed).

## Results

### 1- The effect of GS on the resistance of *E.histolytica* to HS and oxidative stress

**Figure 1:**
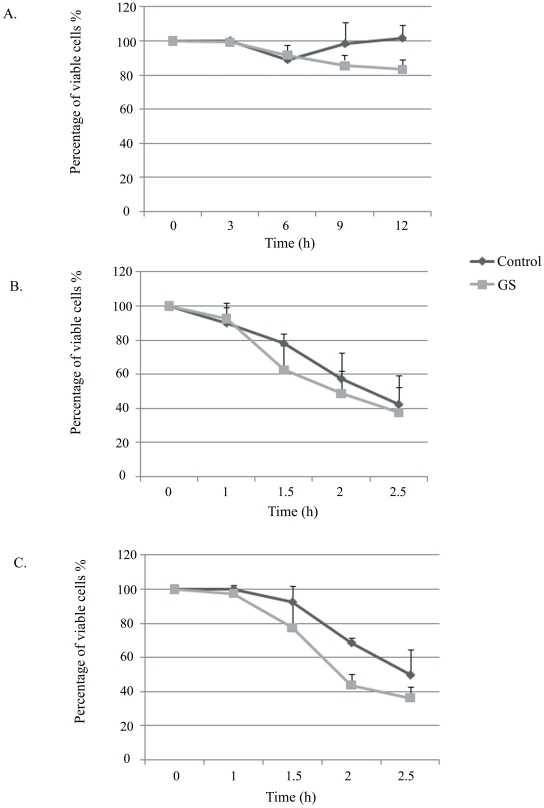
The effect of GS on the resistance of *E.histolytica* to HS and oxidative stress. The viability of trophozoites grown in glucose-free Diamond's TYI-S-33 media for 12 hours (A), subjected to heat shock at 42°C (B) and to oxidative stress (2.5 mM H_2_O_2_) (C) was measured and compared to control trophozoites grown in complete Diamond's TYI-S-33 medium. The number of trophozoites at the beginning of each experiment was taken as 100%. Data are expressed as the mean and standard deviation of three independent experiments that were repeated twice with a P-value <0.05.

*Entamoeba histolytica* trophozoites were glucose-starved for a period of twelve hours and the effect of glucose starvation (GS) on their viability was determined (Fig. 1A). Up to a period of six hours, GS has no significant effect on the viability of the trophozoites. Between 6 hours and 12 hours, a slightly lower viability (17%) was observed for the glucose-starved trophozoites when compared to the viability of parasites cultivated in the presence of glucose. A longer GS (more than 24 hours) led to significant parasite death [18]. Consequently, we used 12 hours of GS for all subsequent experiments.

The induction of stress responses is important for the adaption of cells to environmental changes following stress [28]. Consequently, we examined the resistance of glucose-starved trophozoites to heat shock and to oxidative stress. No significant difference in the resistance of starved and control trophozoites to heat shock was observed (Fig. 1B). In contrast, the glucose-starved parasites were slightly more sensitive to oxidative stress when compared to the sensitivity of control trophozoites grown in presence of glucose (Fig. 1C). In addition, the expression of heat shock protein 70 (HSP70) expression was not up-regulated by GS (Table S1). These results indicate that GS does not induce a protective response against distinct stresses.

### 2- The effect of GS on *E. histolytica* virulence

**Figure 2:**
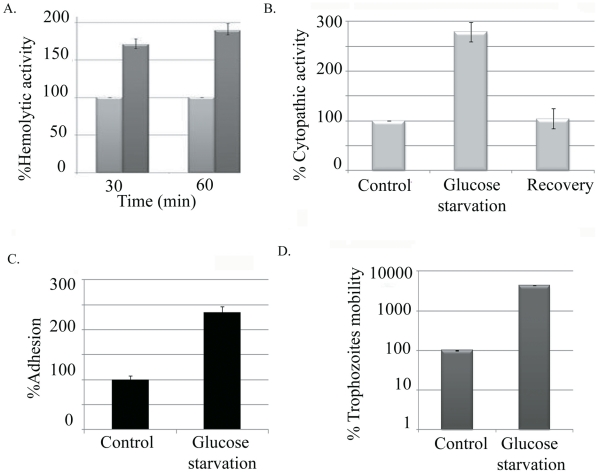
The effect of GS on virulence. The hemolytic activity (A), and the cytopathic activity (B), adhesion (C) and mobility (D) of *E.histolytica* trophozoites were examined. Control cells were grown in complete TYI medium, while the glucose-starved trophozoites were grown in glucose-free TYI for 12 hours. The respective value of the control for each activity was taken as 100%. Data are expressed as the mean and standard deviation of three independent experiments that were repeated twice with a P-value <0.05.

The effect of GS on *E. histolytica* virulence has never been investigated. Since *E. histolytica* trophozoites have the capacity to lyse human erythrocytes [29], we compared the capacity of *E. histolytica* to lyse human erythrocytes [29] (hemolytic activity) of control and glucose-starved trophozoites as one of the measures of their virulence. We found that the hemolytic activity of glucose-starved trophozoites was nearly twice as high (1.7 fold) as that of the control trophozoites (Fig. 2A). Since the parasite is also able to disrupts monolayers of cultured mammalian cells, such as Baby Hamster Kidney (BHK) or HeLa cells [30], we compared the cytopathic activity of control and glucose-starved *E. histolytica* trophozoites as a second measure of virulence. We found that the cytopathic activity of glucose-starved trophozoites was significantly higher than that of the control trophozoites (Fig. 2B). Moreover, the cytopathic activity of the glucose-starved trophozoites can be restored to that of the control trophozoites upon their re-incubation in a glucose-containing culture medium for 12 hours (Fig. 2B). Since the cytopathic process is initiated by the parasite binding to its target cells [31], we then compared the ability of control and glucose-starved *E .histolytica* trophozoites to adhere to HeLa cells. We found that the binding of the glucose-starved trophozoites was more than twice as high as that of the controls trophozoites (Fig. 2C). During invasive amebiasis, migration is an essential process; Therefore, we compared the migration of control and glucose-starved *E. histolytica* trophozoites through 8 µm pores. We found that the numbers of glucose-starved trophozoites that passed through the pores was considerably greater than then number of control trophozoites (Fig. 2D). Collectively, these results indicate that GS is a positive regulator of *E. histolytica* virulence.

### 3- Quantitative proteomic analysis of glucose-starved trophozoites

**Figure 3:**
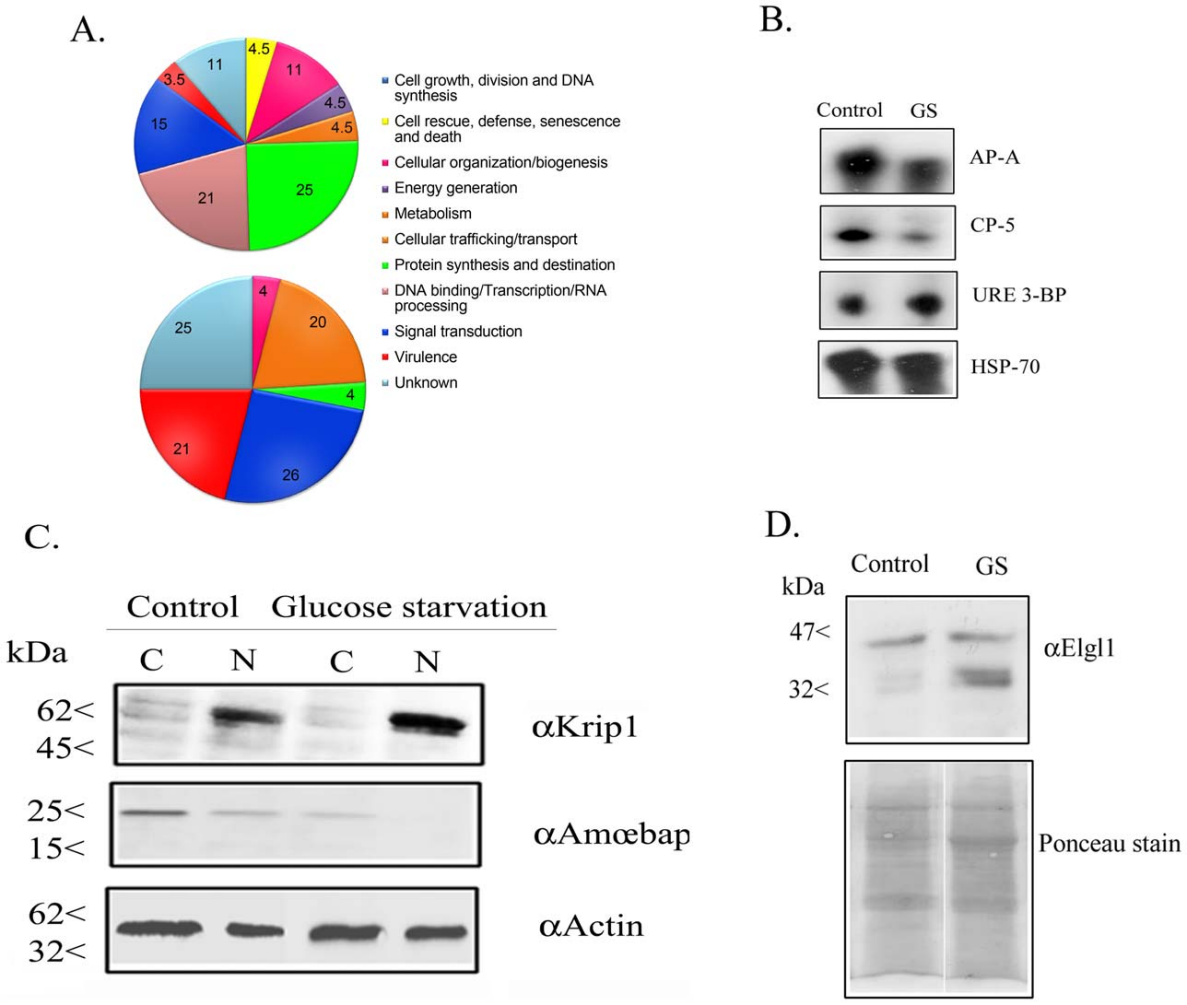
Quantitative changes in protein expression following glucose starvation. (A) Total lysates of control and glucose-starved *E.histolytica* trophozoites used for stable isotope dimethyl labeling of peptides for quantitative proteomics analysis. The pies represent proteins whose amounts were increased (upper panel) or decreased (lower panel) following glucose starvation. The proteins were annotated according to their biological role as classified in the Pfam databases. Percentage number is given for each protein family. (B) Transcript amounts for *Ap-A*, *CP-A5*, *URE-3BP* and *HSP70* in control and glucose-starved (GS) *E.histolytica* trophozoites were analyzed by Northern blot analysis. HSP70 whose expression is only slightly reduced in glucose-starved trophozoites (Table S1) was used as a control for loaded RNA. (C) Western blot analysis of Ap-A and KRiP1 expression in glucose-starved trophozoites. Cytoplasmic and nuclear protein fractions of *E.histolytica* were separated on 12% SDS-PAGE, transferred to a nitrocellulose membrane and incubated respectively with the KRiP1, Ap-A, or actin antibody. (D) Western blot analysis of LgL1 amounts in control and glucose-starved (GS) trophozoites. Membrane protein fractions were separated on 12% SDS-PAGE, transferred to nitrocellulose membrane and incubated with LgL1 antibody. Ponceau stain was used to control for protein loading.

To gain insight into the mechanism by which GS enhances the virulence of *E. histolytica*, we compared changes in the protein composition of control and glucose-starved trophozoites, using stable isotope labeling (Table 2 and 3). In this high throughput proteomics analysis, total protein extracts from *E. histolytica* trophozoites grown in complete TYI media or in GS medium was compared to total lysate from *E. histolytica* grown in GS media (as described in Materials and methods). Proteins were identified using Mass spectrometry (MS) and identification of proteins was achieved by a minimum of three peptides. Forty-nine proteins with at least a two-fold change in their abundance were detected. Of these, 29 proteins were more abundant (Table 2) and 20 proteins were less abundant in the glucose-starved trophozoites when compared to the equivalent proteins in the controls trophozoites (Table 3). We classified the corresponding genes according to their functions using the Pfam classification (Fig. 3A). An interesting feature that we observed for glucose-starved trophozoites was a decrease in the amount of proteins that were associated with metabolism (Fig. 3A; lower panel). These proteins are mostly associated with the fatty acid pathway. The induction of the gluconeogenic pathways is a common response to GS [32]. We found that a protein that shares a short homology to glycerol-3-phosphate dehydrogenase (EHI_161020) was one of the most highly enriched in the glucose-starved trophozoites. An interesting feature of the response to GS is the increase in proteins that are involved in protein synthesis, especially the 60S and 40S ribosomal proteins (Fig. 3A; upper panel). Several other abundant proteins in the glucose-starved trophozoites may play a role in the boosting of *E.histolytica* virulence. One of them is the transcription factor Upstream Regulatory Element 3-Binding Protein (URE3-BP). The enrichment of URE3-BP upon GS treatment correlates with a higher amount of *URE3-BP* transcripts in the glucose-starved parasite compared to control trophozoites (Fig. 3B). URE3-BP is a calcium-responsive transcription factor that is known to modulate the transcription of two *E. histolytica* genes encoding virulence-related factors, the Gal/GalNAc lectin subunit HgL5 and ferredoxin [24]. Further proteins that may play a role in the boosting of *E. histolytica* virulence under GS are proteins encoded by genes up-regulated in trophozoites isolated from hamster liver abscesses [33] such as the 20 kDa antigen (EHI_056490) and the lysine-rich proteins, KRiP1 (EHI_096350) and KRiP3 (EHI_110740) (Table 2 and [33]). The increased abundance of KRiP1 observed in the proteomic analysis was confirmed by Western blot analysis (Fig. 3C). An unexpected result of the proteome analysis is the lower abundance of proteins associated with virulence in the glucose-starved *E.histolytica* trophozoites compared to control trophozoites (Table 3). These proteins include the key virulence factors Ap-A and CP-A5. The lower abundance of Ap-A in glucose-starved trophozoites was confirmed by Western blot analysis (Fig. 3C). This result correlates with a lower amount of *Ap-A* transcripts in the glucose-starved parasites compared to control trophozoites (Fig. 3B). About CP-A5, its lower abundance observed by the proteomic analysis (Table 3) correlates with a lower amount of CP-A5 transcript (Fig. 3B).

**Table 2:** Quantitative proteomic analysis of proteins with increased abundance following glucose starvation.

Accession Pathema	Accession GI	Ppro	pepCount	mean H/L	Protein Name	Domain of interest
EHI_161020	67466970	3.63E-04	3	13.30	hypothetical protein	Glycerol-3-phosphate dehydrogenase
EHI_096350	67483266	2.57E-04	3	12.91	hypothetical protein	Lysine-rich protein KRiP1
EHI_035430	67481531	5.06E-08	6	7.28	hypothetical protein	
EHI_194520	67478606	4.17E-11	7	5.59	hypothetical protein	RRN7/LIM domains
EHI_153080	67464228	3.14E-08	9	5.48	60S ribosomal protein L24	
EHI_114350	67465341	7.49E-05	4	4.79	hypothetical protein	SIR2 family
EHI_156420	67474310	1.91E-09	6	4.34	hypothetical protein	Calponin homology domain
EHI_104600	67464911	4.37E-09	3	4.32	13 kDa ribonucleoprotein-associated protein	
EHI_140650	67477494	2.14E-07	3	3.51	transcription enhancer protein	
EHI_086230	67474712	1.22E-05	3	3.12	hypothetical protein	V-type H+-transporting ATPase subunit G
EHI_092120	67467728	1.14E-05	3	2.96	60S ribosomal protein L44	
EHI_069560	67469807	4.42E-09	8	2.86	hypothetical protein	Autophagy-related protein 27
EHI_110740	67477647	5.28E-06	8	2.83	hypothetical protein	Lysine-rich protein KRiP3
EHI_141930	67478487	1.74E-04	3	2.65	protein kinase	
EHI_114120	67471726	2.28E-10	12	2.51	Chaperonin-containing TCP-1 delta subunit	
EHI_093860	67484130	8.40E-06	3	2.48	MIT-domain protein	
EHI_060740	10505244	6.66E-15	10	2.41	URE3-BP sequence specific DNA binding protein	
EHI_012980	183231113	1.22E-14	21	2.40	dihydropyrimidine dehydrogenase	
EHI_143100	183235312	3.69E-06	5	2.39	Ras guanine nucleotide exchange factor	
EHI_064640	183231981	7.51E-09	6	2.28	hypothetical protein	Seryl-tRNA synthetase N-terminal domain
EHI_020240	67466062	5.21E-04	5	2.27	tryptophanyl-tRNA synthetase	
EHI_000510	67474022	4.36E-06	3	2.27	60S ribosomal protein L4	
EHI_013260	67481903	2.11E-14	4	2.22	Rho family GTPase	
EHI_013890	67463032	1.88E-11	12	2.21	40S ribosomal protein S6	
EHI_124520	183231771	1.13E-06	6	2.21	hypothetical protein	domain Ras-like GTPase superfamily
EHI_056490	67469491	1.23E-06	7	2.08	20 kDa antigen	
EHI_160980	67466177	5.22E-12	24	2.02	methylated LINE binding protein EhMLBP	
EHI_146560	67465753	3.36E-12	8	2.01	40S ribosomal protein S24	
EHI_023230	67480599	5.89E-09	8	2.01	histone H4	

**Table 3:** Quantitative proteomics analysis of proteins with decreased abundance following glucose starvation.

Accession Pathema	Accession GI	Ppro	pepCount	mean H/L	Protein Name	Domain of interest
EHI_001830	183233476	1.70E-07	6	2.00	hypothetical protein	Gpi16 subunit, GPI transamidase component
EHI_109990	67472817	1.05E-09	4	2.00	hypothetical protein	lipoprotein 17
EHI_004610	67483002	7.30E-04	3	2.00	phosphoribulokinase	
EHI_185240	67481639	6.47E-06	6	2.00	long chain fatty acid CoA ligase	
EHI_196530	67465479	2.09E-10	4	2.10	Ras family GTPase	
EHI_121490	67463591	1.65E-09	5	2.10	WD repeat protein	
EHI_146330	67467663	1.44E-14	5	2.20	calpain large subunit domain III-containing protein	
EHI_011550	67474946	4.68E-05	3	2.30	hypothetical protein	Arrestin WW domains
EHI_130930	400978	2.15E-06	5	2.30	Ras-like GTP-binding protein RHO1	
EHI_130930	67480401	3.40E-07	4	2.40	purine nucleoside phosphorylase	
EHI_065740	183234624	7.47E-06	4	2.40	HEAT repeat domain-containing protein	
EHI_065740	67476093	6.55E-14	6	2.40	60S ribosomal protein L30	
EHI_096410	158932	1.52E-08	3	2.60	hypothetical protein	
EHI_118810	67483281	3.40E-04	3	2.00	hypothetical protein	Protein kinase domain
EHI_096410	67477219	2.60E-06	3	2.70	protein kinase domain-containing protein	
EHI_012170	67481683	2.91E-04	3	2.90	hypothetical protein	von Willebrand factor
EHI_127830	67479381	6.51E-05	4	2.90	long chain fatty acid CoA ligase	
EHI_168240	67469932	1.22E-14	5	3.10	cysteine proteinase –A5	
EHI_159480	67475078	1.17E-06	6	4.30	pore-forming peptide ameobapore A precursor	
EHI_194540	183231856	9.24E-08	4	6.30	pore-forming peptide ameobapore B precursor	

Total lysates of control and glucose-starved *E.histolytica* trophozoites were produced and used as substrates for stable isotope dimethyl labeling of peptides for quantitative proteomics analysis. The list describes the proteins that were up-regulated (Table 2) or down-regulated (Table 3) following glucose starvation. Mean H/L provided directly from the MS analysis is the ratio between the protein levels in lysates from glucose-starved and control trophozoites. The value displayed for pepCount represents the number of peptides used for protein identification and statistical value is given in the column Ppro (protein probability). Ppro is the probability of the best peptide match (the peptide with the lowest score) for each identified protein. Classification and characterization of the domains in the hypothetical proteins was done using motif Search (http://motif.genome.jp/) of the PROSITE and Pfam databases.

### 4- Physiological analysis of trophozoites down-regulated for the expression of KRiP1

**Figure 4:**
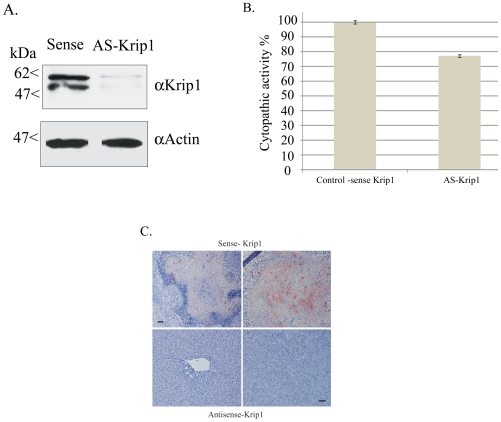
Antisense down-regulation of KRiP1 expression. (A) Determination of *KRiP1* expression in sense KRiP1 and antisense KRiP1 transfectants by Western blot analysis. Nuclear protein fractions of *E.histolytica* sense KRiP1 and antisense KRiP1 transfectants grown in the presence of 40 µg per ml G418 were separated on 12% SDS-PAGE and analyzed by Western blot using a KRiP1 antibody, and an actin antibody. Identical results were obtained for three independent experiments. (B) Cytopathic activity of sense KRiP1 and antisense KRiP1 transfectants grown in complete TYI-S-33 medium. The cytopathic activity of the sense KRiP1 transfectant was taken as 100%. Data are expressed as the mean and standard deviation of three independent experiments that were repeated twice with a P-value <0.05. (C) Amoebic liver abscess formation by *E. histolytica* transfected with sense- and antisense-KRiP1 expression plasmids. Hamsters were infected by intraportal vein injection with trophozoites (1×10^6^) expressing a KRiP1-sense (upper row) or -antisense (lower row) plasmid. A total of 6 animals, in two independent experiments, were infected with each amoeba type. Liver abscess formation was analysed at 7 days post-infection. Images show representative fields from 5 µm sections of formaldehyde-fixed tissue, stained with Harris' haematoxylin, periodic acid-Schiff reagent to visualize the hamster cells. Trophozoites were revealed by monoclonal antibody CD6 directed against *E. histolytica* Gal/GalNAc lectin heavy subunit and HRPO-coupled anti-mouse antibody. Scale bar, 60 µm for upper left panel and 40 µm for the three other panels.

KRiP1 is one of the proteins whose abundance is the most enhanced by GS (Table 2 and Fig. 3C). Moreover, the up-regulation of KRiP1 gene expression in trophozoites isolated from hamster liver abscesses points towards an involvement of this protein in the regulation of *E. histolytica* virulence [33]. These observations suggest an implication of KRiP1 in the mechanism accounting for the exacerbation of amoebic virulence under GS. To test this hypothesis, the expression of KRiP1 was down-regulated using the antisense technology. For this purpose, trophozoites were transformed with a plasmid that includes the *krip1* gene in the antisense orientation between the 5′ and 3′ untranslated regions (UTRs) of the *E. histolytica* gene coding for ribosomal protein RP-L21 (*Ehg*34) [20]–[21]. As a control, trophozoites were transformed with a plasmid that includes the *krip1* gene in the sense orientation. It is important to note that the *Ehg*34 5′ UTR does not allow the translation of an mRNA that has been expressed under its control [34]. The amount of KRiP1 in trophozoites transformed with the *krip1* gene sense or antisense construct was determined by Western blot analysis (Fig. 4A). The KRiP1 antisense strain contained three times less amount of KRiP1 compared with those found in the KRiP1 sense strain. No significant difference in the growth rate of KRiP1 sense and antisense trophozoites cultivated in standard TYI-33 media was observed (data not shown). In addition, no difference in the viability of KRiP1 sense and antisense transformants exposed to GS was observed (data not shown). These results indicate that KRiP1 is not essential for the parasite survival under GS.

**Figure 5:**
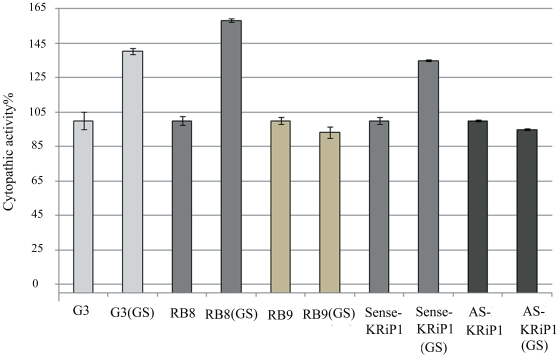
Cytopathic activity under GS of strains with reduced expression of Ap-A, CP-A5, Lgl1, and KRiP1. Cytopathic activity of *E.histolytica* Ap-A mutant (G3), and CP-A5 mutant (RB8), and LgL1 mutant (RB9), and *E.histolytica* sense KRiP1 and antisense KRiP1transfectants that were cultivated in control and glucose-free TYI-S-33 medium. The cytopathic activity of each strain before glucose starvation (GS) was taken as 100%. Data are expressed as the mean and standard deviation of three independent experiments that were repeated twice with a P-value <0.05 (with assays/measurements repeated twice.

We then compared the cytopathic activity of *E. histolytica krip1* sense and antisense trophozoites cultivated in standard TYI-33 media. The cytopathic activity of *E.histolytica* trophozoites expressing the antisense KRiP1 vector was 25% lower compared to that of the control (Fig. 4B). Under conditions of GS, the boosting effect on the cytopathic activity was observed for the KRiP1 sense trophozoites but did not occur for the KRiP1 antisense parasites (Fig. 5). This result suggests that KRiP1 is involved in the mechanism that up-regulated the cytopathic activity of *E. histolytica* under GS. We further analyzed the consequences of *krip1* gene expression levels on the amoebic liver abscess formation by intraportal infection of hamsters with KRiP1 sense or anti-sense trophozoites. Two independent experiments were carried out. Seven days after infection, the animals were sacrificed, morphology of liver was macroscopically observed and a histological analysis was performed. As expected, infection with trophozoites carrying the *krip1* sense gene resulted in abscess development (4 of 6 animals presented well organized abscesses). Histological analysis showed that 7 of the 12 inspected lobes presented necrotic areas with parasites (Fig. 4C). In contrast, trophozoites carrying the *krip1* gene antisense construct have clearly reduced their capacity to establish liver abscesses. Indeed, in 4 out of 6 hamsters, we did not observe macroscopic abscesses. From the 12 lobes examined by histology, only two presented liver lesions, whereas the other lobes presented preserved tissue architecture (Fig. 4C).

### 5- Ap-A and CP-A5 are not necessary for the boosting effect of glucose starvation on virulence

Ap-A and CP-A5 are two major *E. histolytica* virulence factors [35]. The reduction of their levels in glucose-starved trophozoites (Table 3) suggests that these proteins are not involved in the boosting effect of GS on the parasite's virulence. To test this hypothesis, two strains silenced for the expression of Ap-A (G3 strain) and Ap-A and CP-A5 (RB8) were studied. First, we confirmed by RT-PCR that the expression of Ap-A and CP-A5 encoding genes in the respective G3 and RB8 strains was silenced (data not shown). Then, the GS effect on the cytopathic activity of *E. histolytica* strains G3 and RB8 was examined (Fig. 5). We found that GS boosted the virulence of the G3 and RB8 strains indicating that Ap-A and CP-A5 are not requested for the boosting effect of GS on virulence.

### 6- LgL1 is necessary for the boosting effect of glucose starvation on virulence

*E. histolytica* trophozoites adhere to the host colon epithelium using the galactose/N-acetylgalactosamine (Gal/GalNAc) inhibitable lectin [36]. Monoxenic cultivation of *E. histolytica* with the bacterium *Escherichia coli* strain O55 results in down-regulation of the lectin light subunit and reduced virulence [37]. These observations suggest that the Gal/GalNAc lectin light subunits are involved in the response of the parasite to environmental changes. Therefore, and despite the fact that the light subunits were missing from the list of protein regulated by GS (Table 2 and 3), we examined more specifically their abundance in the membrane-enriched protein fraction of glucose-starved trophozoites. We observed that around three times more LgL1 was present in the protein fraction of glucose-starved trophozoites compared to the amount present in the fraction of the control strain (Fig. 3D). To determine the role of LgL1 in the boosting effect of GS on virulence, the cytopathic activity of the *E. histolytica* RB9 strain was studied. These parasites have been silenced for the expression of Ap-A and of LgL1 [38]. Interestingly, no boosting effect of GS on the cytopathic activity of the strain RB9 was observed (Fig. 5). These results suggest that LgL1 is involved in the mechanism that enhances the cytopathic activity of *E. histolytica* under GS.

## Discussion

Parasite survival in its host depends on its ability to react to different stress stimuli and to adapt to changing environments. Under laboratory conditions, *E. histolytica* is cultivated in presence of 1% glucose whereas in its human host, the parasite lives mostly in the large intestine, which is a low*-*glucose environment, due to the absorption of simple sugars in the small intestine [39]. We recently reported evidences that the parasite reacts to GS by modifying the cellular localization of enolase and the methylation status of its tRNAasp [18]. The results presented here indicate that GS is also a positive regulator of *E. histolytica* virulence.

A positive regulation of virulence by GS has also been reported in *Candida albicans*. In this haploid yeast, glucose depletion causes a developmental switch that allows cells to penetrate the surface of an agar medium in a process called invasive growth. This process mimics invasion of host tissue and therefore pathogenesis [40].

In many microorganisms the stress response against heat shock or starvation provides a cross-resistance to other stresses. In *E.coli*, glucose or nitrogen starvation lead to enhanced resistance to heat shock and oxidative stress [41]. Adaptation of *C. elegans* to high temperatures by the heat shock response results in a reduced sensitivity to ethanol under conditions of heat stress [42]. The mechanism acting in the cross-protection effect is the induction of heat shock proteins (HSPs) expression by the initial stress stimulus. HSPs prevent the aggregation of partially denatured proteins [43]. Our results show that GS does not protect the *E. histolytica* against heat shock or oxidative stress. This result is not surprising as the abundance of HSP 70 and other HSPs were not increased in glucose-starved parasites. In contrast to the lack of HSPs induction, GS has a striking effect on the abundance of other *E.histolytica* proteins.

The following focus of our study was directed to identify at the proteome level major *E. histolytica* factors modified by GS. The proteomics analysis provided several candidate(s) proteins that may be involved in the virulence boosting effect of GS.

One of the proteins whose abundance is enhanced in glucose-starved trophozoites is URE3-BP. The role of this transcription factor in the remodeling of the cell surface in response to calcium signals has been well established. Indeed, the expression of a dominant-positive mutant of URE3-BP leads to higher virulence and increased cell motility [24]; phenotypic features that we also observed in glucose-starved trophozoites. These data indicate that URE3-BP may be involved in the control of virulence exerted by GS.

Another putative regulator of the virulence under GS is KRiP1 which belongs to a group of lysine-rich proteins (KRiP and KERP) whose expression is up-regulated in virulent *E.histolytica* strains [33]. Some of these proteins, like KERP1, are localized to the parasite membrane and others like KRiP1 are nuclear, as we demonstrated here. Our knowledge about how these lysine-rich proteins regulate the parasite's virulence is scanty. The fact that no boosting of the cytopathic activity occurs under GS in the KRiP1 antisense transfectants suggests that this protein regulates the virulence of the parasite under stressful physiological conditions.

In an attempt to investigate the direct involvement of KRiP1 in pathogenicity, we subjected parasites modified for KRiP1 production by an antisense strategy to *in vivo* conditions of liver abscess formation. We demonstrate that the antisense parasites exhibit a significantly lower pathogenicity in the hamster model, indicating that KRiP1 plays a role in *E. histolytica* virulence *in vivo*.

A third candidate for the regulation of *E.histolytica* virulence under GS is the Gal/GalNAc lectin light subunit which is also upregulated following GS. The role of LgL1 in the virulence boosting effect of GS may be direct or the result of changes in the overall structure of the Gal/GalNAc lectin complex. In addition, the silencing of LgL1 in the RB9 strain led to the co-silencing of LgL2 and LgL3 [44]. Therefore, it is difficult at this stage to know which LgL is implicated in the GS boosting effect on *E. histolytica* virulence. In *Saccharomyces cerevisiae*, glucose sensing is achieved by two transmembrane glucose-sensing proteins, Snf3 and Rgt2 [45]. These proteins have a cytoplasmic C-terminal tail which is essential for detecting changes in glucose concentrations [46]. In addition to a role of the LgL subunits in adhesion [47], signalling in chemotaxis [48] and differentiation [19], the role of LgL in glucose sensing in *E.histolytica* could be a new area of the Gal/GalNAc lectin research.

An unexpected result of the proteomics analysis is the lower abundance of the virulence factors Ap-A and CP-A5 in glucose-starved *E. histolytica* trophozoites. Usually, in a state of energy depletion organisms synthesize virulence factors that are capable of killing host cells and degrade macromolecules to obtain sugar and energy [49], [50]. Our results suggest that AP-A and CP-A5 are not essential for the virulence boosting in glucose-starved parasites.

An interesting feature that was found in glucose-starved trophozoites is the decreased amount of proteins associated with metabolism and in particular with the fatty acid pathway. In the specific pyruvate-to-ethanol pathway in *E.histolytica*, acetyl-CoA is converted to acetaldehyde, which is then reduced to ethanol [51]. Reduced levels of the long chain fatty acid CoA ligase during GS may be a consequence of a mechanism by which acetyl-CoA is deviated from the fatty acid pathway into the pyruvate-to-ethanol pathway [52]. Lipid degradation as a mean to increase acetyl-CoA production was also observed in glucose-starved *B. subtilis* and in starved rats where fatty acids degradation could provide acetyl-CoA as substrate for the tricarboxylic acid cycle [53], [54]. Many of the ribosomal proteins were also affected by GS. These proteins are regarded as moon light proteins, thus having extra-ribosomal roles [55]. Examples for biological processes ribosomal proteins participate in include growth regulation, cell proliferation and DNA damage response [56], [57], [58]. In eukaryotic cells ribosomal proteins can auto-regulate their production, in this way synthesis of individual ribosomal protein can be controlled [59]. This mechanism may also be relevant for *E. histolytica* for which proteomics data provide evidence for ribosomal turnover during GS and for the accumulation of some several ribosomal proteins [60]. Alternatively, the accumulation of ribosomal components in the glucose-starved parasites may reflect their ability to immediately initiate an upshift program when the missing substrate is made available, as seen in *Vibrio* sp. strain S14 during carbon starvation [61]. At this stage, we cannot rule out that metabolic changes occurring in the glucose-starved parasites contribute to the modulation of their virulence.

In summary, the results of this investigation indicate that significant changes occur in the metabolism of *E.histolytica* exposed to GS to provide the parasite an alternative source of energy. In addition, we report for the first time that GS upregulates *E. histolytica* virulence and that KRiP1 and LgL1 are involved in the boosting effect. In the future, it will be interesting to determine if this specific response of the parasite to short-term glucose starvation reflects events occurring in the low-glucose environment of the large intestine.

## Supporting Information

Table S1 (XLS)
